# Determinants of mortality, intensive care requirement and prolonged hospitalization in malaria – a tertiary care hospital based cohort study from South-Western India

**DOI:** 10.1186/1475-2875-13-370

**Published:** 2014-09-19

**Authors:** Kavitha Saravu, Kumar Rishikesh, Asha Kamath

**Affiliations:** Department of Medicine, Kasturba Medical College, Manipal University, Manipal, 576104 Karnataka India; Department of Community Medicine, Kasturba Medical College, Manipal University, Manipal, India

**Keywords:** *Plasmodium vivax*, *Plasmodium falciparum*, Malaria, Severe malaria, Non-severe malaria, Intensive care, Prolonged hospitalization, Malarial outcomes

## Abstract

**Background:**

There is a remarkable dearth of literature on less pronounced outcomes in malaria, namely prolonged hospitalization and intensive care requirement. Limitations on routine clinical applicability of the World Health Organization’s (WHO) guidelines for determination of severity in malaria does result in underestimation of the true burden of clinicians’ perceived severity in malaria. This study was conducted to evaluate the clinico-laboratory and malarial severity features to determine their association with mortality, prolonged hospitalization and requirement of intensive care outcomes.

**Methods:**

A tertiary care hospital based retrospective study was conducted from the year 2007 to 2011 among microscopically proven adult malaria patients. Logistic regression analysis was performed to determine the factors associated with mortality, more than seven days hospitalization, intensive care and other supportive requirements.

**Results:**

Of a total of 922 malaria cases studied, more than seven days of hospitalization was the most frequent outcome (21.8% (201), 95% CI = 19.1-24.5%) followed by intensive care requirement (8.6% (79), 95% CI = 6.8-10.4%) and in-hospital mortality (1.2% (11), 95% CI = 0.5-1.9%). Odds of mortality were significantly higher with cerebral malaria, pulmonary oedema/acute respiratory distress syndrome (PE/ARDS), liver dysfunction, severe anaemia, renal failure, respiratory distress, metabolic acidosis and leucocytosis. More than seven days hospitalization had inverse association with mortality. *Plasmodium falciparum* infection, more than three days of history of fever, haemoglobinuria, renal failure, shock, leucocytosis, severe thrombocytopaenia and every 10 mmHg fall in systolic blood pressure were the independent predictors of more than seven days of hospitalization. More than three days of history of fever, cerebral malaria, PE/ARDS, renal failure, metabolic acidosis, hyperparasitaemia, leucocytosis and severe thrombocytopaenia were independently associated factors with intensive care requirement.

**Conclusions:**

For routine clinical settings, severity definition for malaria needs to be redefined or modified from the existing WHO guidelines. Leucocytosis and severe thrombocytopaenia should be identified as severity determinant in malaria. Patients having more than three days history of fever, leucocytosis, severe thrombocytopaenia and renal failure are more likely to require either prolonged hospitalization and/or intensive care. PE/ARDS alone in *Plasmodium vivax* may result in mortality, whereas multiorgan involvement is common in *P. falciparum* related mortalities.

**Electronic supplementary material:**

The online version of this article (doi:10.1186/1475-2875-13-370) contains supplementary material, which is available to authorized users.

## Background

Indeed mortality is the most untoward and unfavourable outcome of malaria. Nonetheless, often various progressively worsening clinico-pathophysiological events precede and determine the mortality outcome. The World Health Organization has recognized many such events as clinico-laboratory features of disease severity in malaria [1]. Conspicuously, features of malarial severity determines the choice of anti-malarial regimen, prognosis and other less pronounced outcomes, namely allocation to intensive care facilities and duration of hospitalization. Besides, these less pronounced malarial outcomes in turn become the surrogate determinants of extent of resource utilization and survival/death [2]. Since profile of malarial severity differs extensively among diverse population and endemicity [3], it might also hold true with the determinants of malarial outcomes. Most of the previous studies have described only mortality in malaria with respect to different severity determinants. A few studies [2, 4] have discussed the less pronounced outcomes in *Plasmodium falciparum* malaria. There is no report exclusively describing the less pronounced malarial outcomes together with mortality in adult malaria, except one study [4], in which “unfavourable outcome” was defined as composite of mortality, intensive care requirement, or duration of hospitalization ≥5 days.

Additionally, clinicians often come across patients who do not fulfill standard severity features of malaria but, require anti-malarial regimens recommended for severe cases, supportive lifesaving products, intensive care and/or prolonged hospitalization. Seemingly, the underlying determinants of less pronounced unfavourable outcomes in non-severe malaria remain equivocal. These lacunae in the existing literatures on malarial severity and outcomes warranted an immediate attention and exploration. Current study was aimed to evaluate the clinico-laboratory and malarial severity features to determine their association with mortality, prolonged hospitalization and requirement of intensive care outcomes.

## Methods

### Study design and patients

This study was conducted from January 2007 to December 2011 by extracting patients’ data from their hospital records onto manual pro forma followed by electronic database formation and analysis. Patients’ inclusion criteria were: either gender, ≥18 years, hospitalized with acute malaria, diagnosed by either quantitative buffy coat test or Leishman’s stained peripheral blood smears with the presence of asexual forms of *Plasmodium vivax* or *P. falciparum* or both, with or without gametocytes. Patients with coexistent non-malarial febrile illnesses were excluded. All patients were managed by the hospital clinicians as per their clinical judgement and national guidelines.

### Ethics statement

Before the study commencement, an approval from the institutional ethics committee, Kasturba Hospital, Manipal University, Manipal, Karnataka, India was obtained. Retrospective study design rendered patients’ consent irrelevant; however anonymity of all patients was strictly maintained.

### Variables

#### Independent variables

Malaria severity was defined as per the World Health Organization’s (WHO) guidelines for the management of severe falciparum malaria in the year 2012 [1]. An adaptation in clinical jaundice/liver dysfunctions’ definition was made as rise in total bilirubin ≥2.5 mg/dL with simultaneous three fold elevation in any serum aminotransferases from their reference upper limits. Severity determinants were restricted to cerebral malaria (impaired consciousness, coma or multiple generalized convulsions within 24 hours), liver dysfunction, pulmonary edema (PE) or acute respiratory distress syndrome (ARDS) (radiological), renal failure (serum creatinine >3 mg/dL), shock (systolic blood pressure <80 mm Hg), spontaneous bleeding, hyperparasitaemia (parasite index >5%, i.e. percentage of parasitized erythrocytes on Leishman’s stained peripheral blood smear), hypoglycaemia (blood sugar <40 mg/dL), respiratory distress (respiratory rate >32 beats/minute), metabolic acidosis (plasma bicarbonate <15 mmol/L) and severe anaemia (hemoglobin <7 g/dL). In addition, routine clinico-laboratory parameters and supportive requirements were also included as independent variables. The severity determinants were the worst parameters during hospitalization as per the WHO guidelines [1].

#### Dependent variable

In-hospital mortality, intensive care requirement and duration of hospitalization were the primary outcomes.

### Statistical analysis

Dichotomous variables were summarized as frequency and proportions by malaria species. Logistic regression analyses were performed to determine the factors associated with mortality, more than seven days hospitalization, intensive care and supportive requirements across malaria cohort, it’s severe and non-severe subgroups. Variables which yielded a p-value ≤0.2 in univariate logistic regression analysis were further analysed through multivariate logistic regression model by ‘forward-Wald’ method. All tests of significance were two-sided, with a p-value of <0.05 indicating statistical significance. Data analysis was done using Statistical Package for the Social Sciences version 15.0 (SPSS, South Asia, Bangalore, India).

## Results

**Figure 1:**
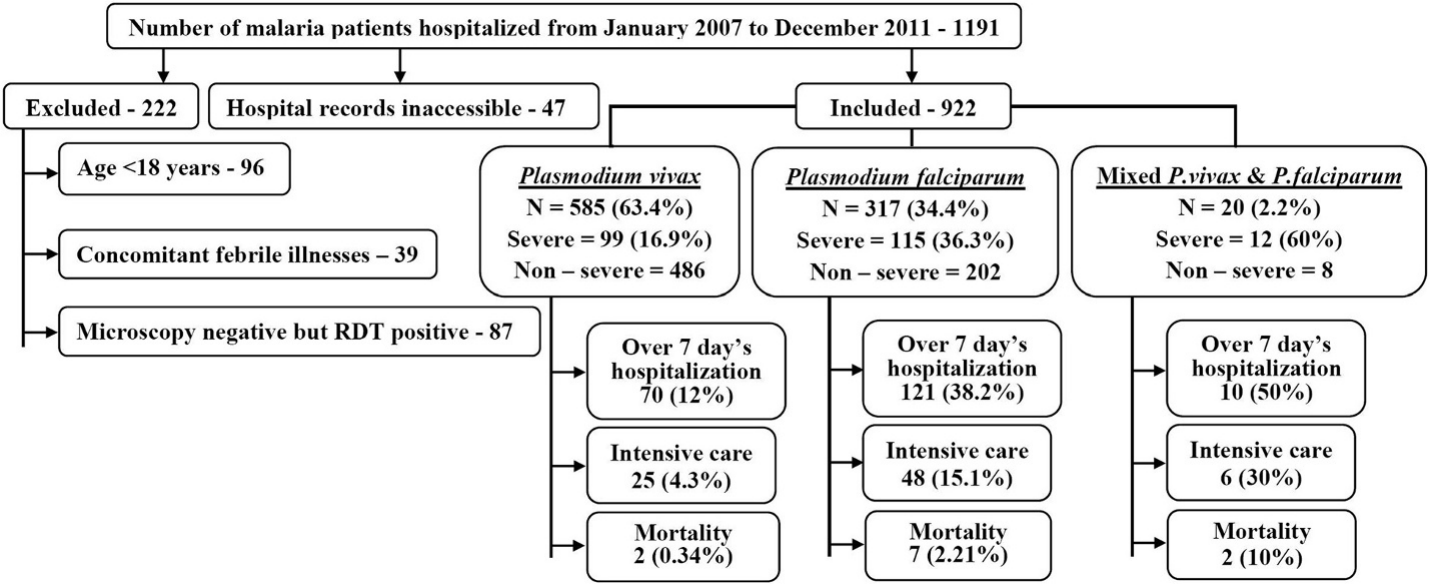
**Flow chart of patients’ selection and distribution of study outcomes across malaria species.**

Of a total of 922 malaria patients studied, *P. vivax* was the largest (63.4%, 95% confidence interval (CI) = 60.3-66.5%) infecting species followed by *P. falciparum* (34.4%, 95% CI = 31.3-37.5%) and their mixed infections (2.2%, 95% CI = 1.3-3.2%), Figure 1. Severe malaria was noted among 24.5% (226/922) patients, 95% CI = 21.7-27.3%. Occurrences of various complications and their combinations in the present study have been reported previously [5]. Among study outcomes, more than seven days of hospitalization was most frequent (21.8%, 95% CI = 19.1-24.5%) followed by intensive care requirement (8.6%, 95% CI = 6.8-10.4%) and in-hospital mortality (1.2%, 95% CI = 0.5-1.9%), Figure 1.

### Factors associated with mortality in complicated malaria

**Figure 2:**
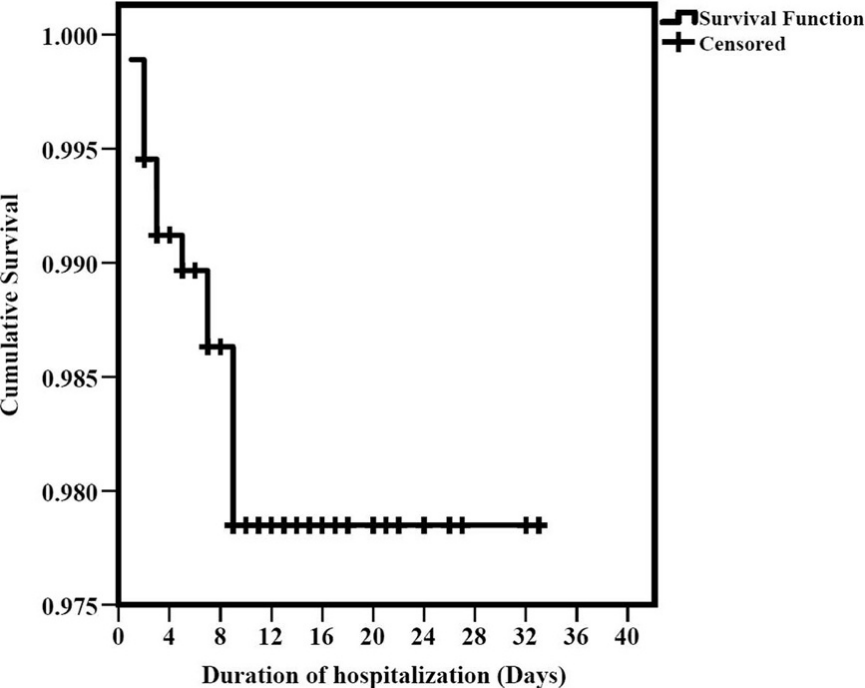
**Kaplan – Meier plot showing cumulative survival probability during hospitalization.**

Mortality occurred with 2 (2%), 7 (6.1%) and 2 (16.7%) severe *P. vivax*, *P. falciparum* and their mixed infections, respectively. A binary logistic regression analysis was performed among severe cohort of 226 patients to determine the factors associated with mortality (Table 1). Though, mortality proportion was higher in *P. falciparum* than *P. vivax* it was not significant statistically (P = 0.16). Of severity determinants, odds of mortality were significantly higher with cerebral malaria, PE/ARDS, liver dysfunction, severe anaemia, renal failure, respiratory distress and metabolic acidosis. Leucocytosis was also found to be associated with mortality. Among supportive requirements, inotropes, mechanical ventilation, dialysis and intensive care were associated with mortality. However, more than seven days of hospitalization had inverse association with mortality [odds ratio (95% confidence interval), 0.11(0.0-0.91)], as the probability decreased with time after admission, Figure 2.

**Table 1:** **Factors associated with mortality in complicated malaria (N = 226)**

Risk factors	Risk factors, % (event/226)	Mortality per risk factor, % (death/no. of event)	Odds ratio [95% confidence interval]*	P - value*
***Species of infection*** ^**$**^				
*Plasmodium vivax*	43.8 (99)	2 (2/99)	Reference	
*Plasmodium falciparum*	50.9 (115)	6.1 (7/115)	3.14 [0.64 - 15.50]	0.20
***Age***				
Up to 40 years	58 (131)	4.6 (6/131)	Reference	
Between 41–60 years	35 (79)	6.3 (5/79)	1.41 [0.42 - 4.77]	0.58
More than 60 years	7 (16)	0	0	0.99
***Gender***				
Female	22.6 (51)	3.9 (2/51)	Reference	
Male	77.4 (175)	5.1 (9/175)	0.75 [0.16 - 3.60]	0.72
***Determinants of severity***				
Cerebral malaria	11.1 (25)	16 (4/25)	**5.28 [1.43 - 19.53]**	**0.01**
Spontaneous bleeding	31.4 (71)	7 (5/71)	1.88 [0.55 - 6.38]	0.31
Pulmonary oedema/ARDS	28.3 (64)	10.9 (7/64)	**4.85 [1.37 - 17.19]**	**0.01**
Liver dysfunction	20.8 (47)	12.8 (6/47)	**5.09 [1.48 - 17.50]**	**0.01**
Severe anaemia	15 (34)	14.7 (5/34)	**5.35 [1.53 - 18.65]**	**0.01**
Haemoglobinuria	11.1 (25)	8 (2/25)	1.85 [0.38 - 9.07]	0.45
Renal failure	21.2 (48)	14.6 (7/48)	**7.43 [2.08 - 26.57]**	**0.002**
Respiratory distress	17.7 (40)	12.5 (5/40)	**4.29 [1.24 - 14.82]**	**0.02**
Metabolic acidosis	5.8 (13)	23.1 (3/13)	**7.69 [1.77 - 33.47]**	**0.01**
Hyperparasitaemia	4.4 (10)	10 (1/10)	2.29 [0.26 - 19.87]	0.45
Hypoglycaemia	0.9 (2)	100 (2/2)	#	#
Shock	4.9 (11)	0	#	#
***Other abnormal lab parameters***				
Leucocytosis (≥11,000/mm3)	15 (34)	17.6 (6/34)	**9.38 [2.49 - 35.33]**	**0.001**
Leucopaenia (≤4,000/mm3)	11.5 (26)	3.8 (1/26)	0.79 [0.10 - 6.51]	0.83
Severe thrombocytopaenia (≤40,000/mm3)	38.1 (86)	8.1 (7/86)	2.73 [0.77 -9.61]	0.12
***Supportive requirements***				
Inotropes	8 (18)	38.9 (7/18)	**32.46 [8.25 - 127.73]**	**<0.001**
Blood transfusion	36.7 (83)	7.2 (6/83)	2.15 [0.64 - 7.28]	0.22
Mechanical ventilation	12.4 (28)	32.1 (9/28)	**46.42 [9.35 - 230.60]**	**<0.001**
Dialysis	10.2 (23)	17.4 (4/23)	**5.90 [1.58 - 21.97]**	**0.01**
Intensive care	30.1 (68)	14.7 (10/68)	**27.07 [3.39 - 216.15]**	**0.002**
**Length of hospital stay**				
More than 7 days of hospital stay	44.7 (101)	1 (1/101)	**0.11 [0.01 - 0.91]**	**0.04**

*Confidence intervals that do not overlap the null value of odds ratio = 1, and P-value <0.05 are shown in bold font.

^#^Statistics could not be computed.

^**$**^Mixed infection was excluded due to insufficient cohort size.

### Factors associated with duration of hospitalization

Duration of hospitalization was dichotomized by more than seven days of hospital stay. Further, the association of more than seven days of hospitalization with independent clinico-laboratory variables among malaria cohort, severe and non-severe subgroups of malaria cohort was analysed separately. In the malaria cohort, *P. falciparum* infection, more than three days of history of fever, haemoglobinuria, renal failure, shock, leucocytosis and severe thrombocytopaenia were the independent predictors of more than seven days of hospitalization (Table 2).

**Table 2:** **Factors associated with more than 7 days of hospital stay^ (Malaria cohort = 922)**

Risk factors	>7 days hospitalization % (event/total number)	Odd ratio [95% confidence interval]*	P - value*	Adjusted odd ratio [95% confidence interval]*	P - value*
**Species of infection** ^**$**^					
*Plasmodium vivax*	12 (70/585)	Reference		Reference	
*Plasmodium falciparum*	38.3 (121/316)	**4.57 [3.26 - 6.40]**	**<0.001**	**3.15 [2.16 - 4.60]**	**<0.001**
**Age**					
Up to 40 years	18.4 (115/626)	Reference			
Between 41–60 years	30.2 (75/248)	**1.99 [1.41 - 2.81]**	**<0.001**		
More than 60 years	22.9 (11/48)	1.39 [0.69 - 2.81]	0.36		
**Gender**					
Male	20.8 (158/760)	1.37 [0.93 - 2.03]	0.11		
Female	26.5 (43/162)	Reference			
**More than 3 days of fever**	25.9 (165/638)	**2.62 [1.74 - 3.95]**	**<0.001**	**2.12 [1.33 - 3.39]**	**0.002**
**Determinants of severity**					
Cerebral malaria	60 (15/25)	**5.73 [2.53 - 12.95]**	**<0.001**		
Spontaneous bleeding	42.3 (30/71)	**2.91 [ 1.76 - 4.79]**	**<0.001**		
Pulmonary oedema/ARDS	43.8 (28/64)	**3.08 [1.83 - 5.18]**	**<0.001**		
Liver dysfunction	51.1 (24/47)	**4.11 [2.27 - 7.45]**	**<0.001**		
Severe anaemia	50 (17/34)	**3.81 [1.91 - 7.61]**	**<0.001**		
Haemoglobinuria	72 (18/25)	**10.06 [4.14 - 24.45]**	**<0.001**	**4.69 [1.63 - 13.56]**	**0.004**
Renal failure	62.5 (30/48)	**7.26 [ 3.91 - 13.46]**	**<0.001**	**2.20 [1.06 - 4.55]**	**0.035**
Respiratory distress	32.5 (13/40)	1.78 [0.90 - 3.51]	0.10		
Metabolic acidosis	61.5 (8/13)	**5.93 [1.92 - 18.32]**	**0.002**		
Hyperparasitaemia	40 (4/10)	2.42 [0.68 - 8.65]	0.18		
Hypoglycaemia	0	#	#	#	#
Shock	54.5 (6/11)	**4.40 [1.33 - 14.57]**	**0.02**	**4.88 [1.17 - 20.42]**	**0.03**
**Other abnormal lab parameters**					
Leucocytosis (≥11,000/mm3)	53.1 (26/49)	**4.59 [2.54 - 8.30]**	**<0.001**	**2.29 [1.13 - 4.64]**	**0.02**
Leucopaenia (≤4,000/mm3)	20.7 (35/169)	0.89 [0.59 - 1.34]	0.57		
Severe thrombocytopaenia (≤40,000/mm3)	37.3 (76/204)	**0.36 [0.25 - 0.51]**	**<0.001**	**1.93 [1.29 - 2.88]**	**0.001**

*Confidence intervals that do not overlap the null value of odds ratio = 1, and P-value <0.05 are shown in bold font.

^#^Statistics could not be computed.

^^^Patients with mortality outcome were excluded from analysis.

^**$**^Mixed infection was excluded due to insufficient cohort size.

In severe subgroup, haemoglobinuria [adjusted odds ratio (95% confidence interval), 3.0 (1.1-8.3)], renal failure [2.4 (1.1-4.8)] and leucocytosis [2.6 (1.1-6.0)] were the independent predictors of more than seven days of hospitalization. However severe *P. falciparum* infection showed significantly higher odds [2.2 (1.3-3.8)] with respect to severe *P. vivax* for more than seven days hospital stay on univariate logistic regression but, it did not figure in the multivariate logistic regression analysis.

In non-severe subgroup, *P. falciparum* infection [7.6 (1.4 – 42.4)] and every 10 mmHg fall in systolic blood pressure [0.6 (0.3-0.9)] were the independent predictors of more than seven days of hospitalization.

### Factors associated with intensive care support

In malaria cohort, over three days of history of fever, cerebral malaria, PE/ARDS, renal failure, metabolic acidosis, hyperparasitaemia, leucocytosis and severe thrombocytopaenia were independently associated with intensive care (Table 3). In severe subgroup, >3 days of history of fever [5.4 (1.3-21.9)], PE/ARDS [3.3 (1.5-7.3)], metabolic acidosis [12.3 (1.8-82.8)], leucocytosis [5.1 (2.0-13)] and severe thrombocytopaenia [3.5 (1.6-7.5)] were the independent predictors of intensive care. *P. falciparum* was more likely to result in intensive care need in both the malaria cohort [4.0 (2.41-6.62)] and the severe malaria subgroup [2.1 (1.1-3.8)] in univariate logistic regression analysis. Intensive care support was needed among substantially less proportion i.e. 1.6% (11/696) of non-severe subgroup.

**Table 3:** **Factors associated with intensive care support^ (Malaria cohort = 922)**

Risk factors	Intensive care support % (event/total number)	Odds ratio [95% confidence interval]*	P – value*	Adjusted odds ratio [95% confidence interval]*	P – value*
**Species of infection** ^**$**^					
*Plasmodium vivax*	4.3 (25/585)	Reference			
*Plasmodium falciparum*	15.1 (48/317)	**4.00 [2.41 - 6.62]**	**<0.001**		
**Age**					
Up to 40 years	6.2 (39/626)	Reference			
Between 41–60 years	12.9 (32/248)	**2.23 [1.36 - 3.65]**	**0.001**		
More than 60 years	16.7 (8/48)	**3.01 [1.32 - 6.87]**	**0.01**		
**Gender**					
Male	7.8 (59/760)	1.67 [0.98 - 2.87]	0.06		
Female	12.3 (20/162)	Reference			
**More than 3 days of fever**	11.1 (71/638)	**5.55 [2.38 - 12.94]**	**<0.001**	**5.87 [1.56 - 22.10]**	**0.01**
**Determinants of severity**					
Cerebral malaria	64 (16/25)	**25.53 [10.00 - 55.39]**	**<0.001**	**12.79 [2.94 - 55.71]**	**0.001**
Spontaneous bleeding	36.6 (26/71)	**8.70 [4.98 - 15.19]**	**<0.001**		
Pulmonary oedema/ARDS	46.9 (30/64)	**14.57 [8.24 - 25.74]**	**<0.001**	**9.44 [4.07 - 21.88]**	**<0.001**
Liver dysfunction	40.4 (19/47)	**9.22 [4.87 - 17.46]**	**<0.001**		
Severe anaemia	29.4 (10/34)	**4.93 [2.27 - 10.74]**	**<0.001**		
Haemoglobinuria	52 (13/25)	**13.83 [6.07 - 31.54]**	**<0.001**		
Renal failure	47.9 (23/48)	**13.44 [7.17 - 25.17]**	**<0.001**	**4.70 [1.74 - 12.69]**	**0.002**
Respiratory distress	32.5 (13/40)	**5.95 [2.93 - 12.08]**	**<0.001**	**5.50 [1.70 - 17.85]**	**0.005**
Metabolic acidosis	76.9 (10/13)	**40.58 [10.91 - 150.90]**	**<0.001**	**10.00 [1.26 - 79.57]**	**0.03**
Hyperparasitaemia	40 (4/10)	**7.44 [2.05 - 26.95]**	**0.002**	**5.69 [1.05 - 30.74]**	**0.04**
Hypoglycaemia	50 (1/2)	10.42 [0.65 - 168.44]	0.10	#	#
Shock	36.4 (4/11)	**6.37 [1.82 - 22.25]**	**0.004**		
**Other abnormal lab parameters**					
Leucocytosis (≥11,000/mm3)	46.9 (23/49)	**13.92 [7.41 - 26.16]**	**<0.001**	**4.17 [1.54 - 11.24]**	**0.005**
Leucopaenia (≤4,000/mm3)	2.4 (4/169)	**0.23 [0.08 - 0.63]**	**0.004**	0.26 [0.07 - 1.00]	0.05
Severe thrombocytopaenia (≤40,000/mm3)	23.5 (48/204)	**7.33 [4.43 - 12.12]**	**<0.001**	**5.70 [2.77 - 11.73]**	**<0.001**

*Confidence intervals that do not overlap the null value of odds ratio = 1, and P-value <0.05 are shown in bold font.

^#^Statistics could not be computed.

^^^Patients with mortality outcome were excluded from analysis.

^**$**^Mixed infection was excluded due to insufficient cohort size.

### Association of >7 days of hospitalization, intensive care and malaria species with supportive requirements

**Figure 3:**
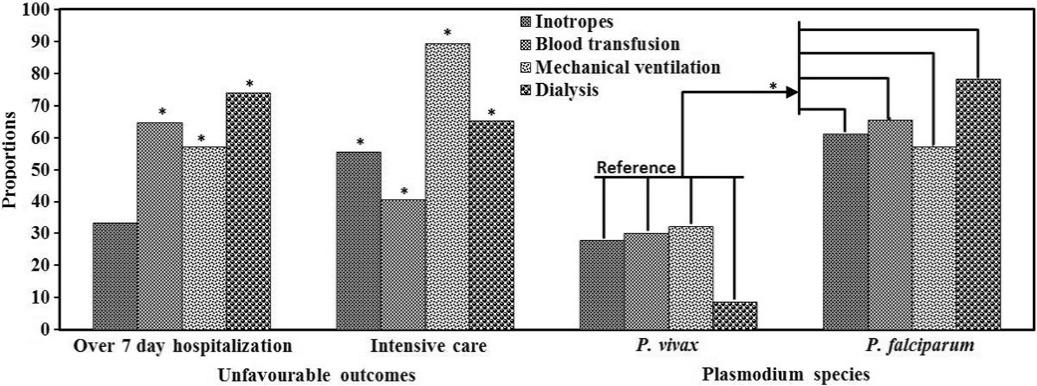
Association of more than 7 days of hospitalization, intensive care requirement and malaria species with supportive requirements. *p <0.05.

On univariate logistic regression analysis, patients with over seven days of hospitalization had significantly higher likelihood of blood transfusion [9.96 (6.46 - 15.34)], mechanical ventilation [5.10 (2.37 - 10.96)] and dialysis [11 (4.28 - 28.28)] in malaria cohort. Likewise, patients with intensive care support were significantly more prone to have inotrope [15.13 (5.78 - 39.57)], blood transfusion [16.15 (9.68 - 26.94)], mechanical ventilation [129.48 (37.89 - 442.42)] and dialysis requirements [24.46 (10.00 - 59.87)]. On inter-species comparison, *P. falciparum* showed significantly higher odds for inotrope [4.17 (1.44-12.11)], blood transfusion [4.94 (3.20-7.61)], mechanical ventilation [3.40 (1.48-7.78)] and dialysis [17.55 (4.05-76.13)] than *P. vivax* (Figure 3).

## Discussion

Global health care diligence intends to avert malaria exclusively. However, its untoward occurrences and aggravations invariably results in extensive resource utilization to try reverting the ailment. Aftermaths of malaria, namely prolonged hospitalization, intensive care requirements and most unfortunate ‘mortality’ , are the sheer reflection of underlying disease severity and extensive resource utilizations. Here, few determinants of these unpleasant outcomes in adult malaria at a tertiary care setting have been identified. Considering clinico-pathophysiological parameters as the independent determinants of the disease severity and the outcomes, those were analysed across malaria cohort, severe and non-severe subgroups of total cohort.

Logistic regression analysis revealed significant association with mortality of few typical and critical complications in severe malaria. Noticeably, occurrences of these complications often results in intensive care requirements comprising management with inotropes, mechanical ventilation, dialysis requirements, blood transfusion etc. With mortality outcome, association of cerebral malaria [6–9], PE/ARDS [6, 8, 9], liver dysfunction [9, 10], severe anaemia [7, 10], renal failure [6, 8, 10], respiratory distress [7, 8, 10], metabolic acidosis [9, 10] and leucocytosis [11] have been reported in various other series as well. Occurrence of leucocytosis is inconsistent in all forms of malaria and depends on factors, such as severity of disease, parasitaemia, host immunity and concomitant infections [12]. However, none of the study patients were having bacteraemia or concurrent infections, as this was an exclusion criterion of this study. Thus, either bacteraemia or concurrent infection as a cause of leucocytosis is overruled. Furthermore, no history of malaria in cases of mortality except one (see Additional file 1) suggests non-immune status and vigorous inflammatory response resulting in baseline leucocytosis [13]. However, specific immunologic factors and their association with disease severity/outcomes in apparently non-immune versus immune malaria patients with either patent or multiple/recurrent infections needs to be determined through a prospective approach. Of severity features leading to mortality, both the *P. vivax* infected mortality was due to PE/ARDS and respiratory distress only, whereas other mortalities were due to multiorgan complications (Additional file 1).

Consistently across the groups, *P. falciparum* infection itself was found to be the independent predictor of more than seven days hospitalization. This appears to be due to higher severity proportion of *P. falciparum* than *P. vivax*. Further, more than three days of history of fever gives an inference of delayed presentation/diagnosis/onset of treatment, which would increase the probability of developing complications [14], thereby prolonged hospital stay and/or intensive care requirement/s. Hemoglobinuria, renal failure and shock are few of the pre-determined and recognized severity determinants by the WHO [1], thus prolonged hospitalization in their occurrence appears to be an obvious outcome. Leucocytosis and severe thrombocytopaenia showing an independent association with more than seven days of hospitalization, intensive care requirement and mortality appears to be a reliable and cost effective laboratory measure, which qualifies to be identified as a standard and uniform determinant of severity in malaria. However, further exploration to validate the significance of leucocytosis and thrombocytopaenia in well planned large cohort is certainly warranted in other settings and regions. Thrombocytopaenia having strong association with prolonged duration of hospitalization, increased intensive care requirement and increased mortality has also been reported previously [4]. Both the less pronounced outcomes of this study have been observed in substantial proportions among non-severe malaria cohort as well (Figure 1).

A proportion of non-severe subgroup staying longer than seven days in hospital is actually a reflection of the course of artesunate based combination treatment given for all *P. falciparum* cases. Additionally, this also appears to be a result of physicians’ perceived severe malaria diagnosis and intensive management in view of either thrombocytopaenia or mild hypotension or other pernicious features not fulfilling the standard severity criteria. This is being emphasized here that, for routine clinical practice, strict adoption of WHO defined severity criteria seems inadequate due to its inherent research oriented limitations [3, 4].

Noticeably, insignificant mortality observed in present cohort can be explained in the light of substantially high proportion of patients, even “non-severe” malaria patients getting either intensive care or other supportive requirements, or prolonged hospitalization. Thus, it has been substantiated that, by clinical practice oriented severity definitions and intensive management worse outcomes in malaria can be minimized in routine clinical settings. Therefore, either revision of existing WHO severity guidelines or development of new severity guidelines exclusively for clinical practice is required.

Perhaps, this is the first exclusive study describing outcomes of malaria in adults. Sufficiently large cohort and robust statistical analysis imparts significant strength to the study findings. Furthermore, stratified evaluation of factors for their association with outcomes across malaria cohort, severe and non-severe subgroups confers additional visibility. Retrospective approach imposes certain limitations to this study. Data on concurrent non-febrile comorbidities were not captured for this study, thus their additive effects on the study outcomes could not be deduced. Malaria diagnosis and speciation was solely based on microscopy and lacked molecular confirmation. Substantially lesser mortality events restricted a legitimate multivariate logistic regression analysis. Thus, future studies on malarial outcomes must comprise sufficient subjects each with and without the study outcomes to determine more robust association. Importantly, a multicentric study approach would be greatly useful to assess and determine the extent of subjectivity associated with criteria for hospitalization and intensive unit care across hospitals within different regions.

## Conclusions

For routine clinical settings, severity definition for malaria needs to be redefined or modified from the existing WHO guidelines. Leucocytosis and severe thrombocytopenia should be identified as severity determinant in malaria and validated through prospective approach to determine a reliable intercept. Further, this study affirms *P. falciparum* infection to be an independent predictor of prolonged hospitalization. Patients having more than three days history of fever, leucocytosis, severe thrombocytopaenia and renal failure are more likely to require either prolonged hospitalization and/or intensive care. Additionally, haemoglobinuria and shock are the independent predictors of prolonged hospitalization, whereas, cerebral malaria, PE/ARDS, respiratory distress, metabolic acidosis and hyperparasitaemia are the independent predictors of intensive care requirement in malaria. PE/ARDS alone in *P. vivax* may result in mortality, whereas multiorgan involvement is common in *P. falciparum* related mortalities.

## Electronic supplementary material

Additional file 1: **Profile of patients with mortality outcome.** The clinico-laboratory data of all patients with mortality outcome have been depicted. (XLSX 15 KB)
